# Seroprevalence of *Taenia saginata* Cysts in Cattle, Human Hospital Cases, and Risk Factors for Human Taeniasis in Kajiado County, Kenya

**DOI:** 10.1155/2023/7385643

**Published:** 2023-10-18

**Authors:** Ruphline M. Anyango, Timothy M. Wachira, Gerald M. Muchemi

**Affiliations:** Department of Public Health, Pharmacology and Toxicology, University of Nairobi, Nairobi, Kenya

## Abstract

*Taenia saginata* is a zoonotic tapeworm that causes diseases in cattle and humans. A cross-sectional survey was conducted between June and July 2021 in Kajiado County, Kenya, to estimate the seroprevalence of *Taenia saginata* cysts in cattle presented for slaughter in Kitengela, Kiserian, and Keekonyokie slaughterhouses; determine the annual hospital reported cases of *T. saginata* in humans that visited all level 4 hospitals in Kajiado County between 2015 and 2021; and assess the risk factors for *Taenia saginata* in humans. Analyzed data in this study revealed that the seroprevalence of *Taenia saginata* cysts in cattle in the selected slaughterhouses was 2.67% (4/150). The meat inspectors recorded no cysts during the study period, confirming that antibody ELISA is three times as sensitive as meat inspection. Data from hospital records showed that a total of 1,487,687 patients visited different facilities in Kajiado County between 2015 and 2021. During the same period, 29 patients were diagnosed with human taeniasis. From the risk factors assessed, uninspected home slaughter (75%), consumption of raw/improperly cooked beef (100%), and open defecation by herders (11%) still pose a risk to humans getting infected by *Taenia saginata*, while the presence and use of latrines (89%) and availability, accessibility, and use of taenicides (89%) seem to have significantly contributed to the reduction of *Taenia saginata* prevalence in this region. It is recommended that control of the infection should be centered towards continuous focused education coupled with regular deworming of the herders and school going children. This will gradually result in behavior and culture change that will ultimately reduce the prevalence and lead to the elimination of the disease.

## 1. Introduction/Background

*Taenia saginata*, the beef tapeworm, is an important zoonotic parasite that causes human taeniasis and bovine cysticercosis. The tapeworm has the cattle as the primary intermediate host and the buffalo, giraffe, and llama as alternative intermediate hosts [1] and the human as the definitive host. The tapeworm causes substantive losses in the meat sector and in particular, the beef industry. Humans get infection when they ingest raw or undercooked beef that contain viable cysticerci while the cattle get infected when they ingest pasture or drink water that is contaminated with parasite egg [2]. The disease cycle is maintained when an infected human releases proglottids by defecating in the open, by active migration of proglottids, when the livestock feed on contaminated pasture or fodder, or when infected persons handle cattle or their feeds [3].

A high prevalence between 10% and 80% has been recorded in Africa, in particular Eastern and Southern African countries including Kenya, Ethiopia, Zambia, South Africa, Zimbabwe, Sudan, and Botswana [4] due to breach of sanitary measures and because most small scale households keep bovine as a source of livelihood, source of food, and source of manure [4].

The disease causes economic losses due to reduced productivity, trade restrictions, downgrading, and condemnation of organs or the entire carcass in the abattoir depending on the number of cysts [5]. These losses are usually quantified in terms of market price of cattle, cost of treatment of an infected carcass, cost of treatment of an infected person, disease prevalence, and grading of the infected carcass [5]. In humans, taeniasis causes fear and distress as a result of pruritus, nausea, abdominal discomfort, weight loss, and mild diarrhea [4]. Serious gastrointestinal tract disorders such as intestinal blockage and peritonitis have also been reported [6].

Diagnosis of *Taenia saginata* in cattle is usually done at meat inspection in the abattoir. This follows a standard as recommended by the Meat Control Act of Kenya, of visual inspection, palpation, and incision of the predilection sites [7]. Routine meat inspection has been reported to underestimate prevalence rates, according to previous study [8] and the most recent study in Belgium [9, 10]. Serological diagnostic tools which detect either antigens or antibodies have since been developed and used for detection of *Taenia saginata* [11–13].

To control human taeniasis and bovine cysticercosis, it is recommended that beef from an infected cattle is cooked properly at temperatures between 56°C and 65°C to ensure all cysts are killed and the meat is safe [14]. Other control measures include the use of drugs to treat humans that have presented with typical signs of taeniasis, that is, ridding off the proglottids. The medication includes praziquantel, tribendimidine, albendazole, and niclosamide [15]. Other measures include community education on use of latrines, consumption of properly cooked beef, and inspection of carcass before release for human consumption[16].

*Taenia saginata* directly and indirectly impacts livestock keeping, which is the main economic activity of residents of Kajiado County, Kenya. A most recent study [17], using meat inspection, revealed moderately high prevalence of bovine cysticercosis and human taeniasis and recommended the use of serological and molecular techniques for advanced diagnosis.

Previous studies [8–10, 12] have relied on meat inspection which has been proven to underestimate by 50% prevalence rates [18]. Meat inspection can only detect the heavily infected carcasses that have the cysts. Furthermore, meat inspectors only rely on predilection sites to detect cysts and can easily miss out on cysts in other areas that are not predilection sites [19]. It has been shown that ELISA is three times more sensitive than meat inspection and should be used for epidemiological studies [18].

The aim of this study was to determine the seroprevalence of *C. bovis* in Kajiado County; the hospital reported cases of human taeniasis over a period of six years and risk factors associated with *Taenia saginata* cysticercosis and taeniasis in Kajiado County, Kenya. This was a mixed study model. The findings of this study would be used to provide epidemiological data for designing prevention and control strategies that are suitable for Kajiado County.

## 2. Materials and Methods

### 2.1. Study Area

**Figure 1:**
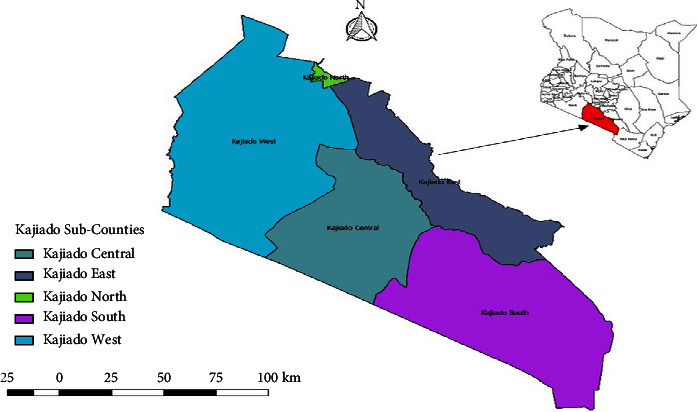
Map of Kenya showing administrative regions in Kajiado County.

The study area selected was Kajiado County, as shown in Figure 1. The study area was purposively selected because the main economic activity is livestock rearing (Kajiado County Integrated Development Plan, 2018–2022), and cases of *Taenia saginata* have been reported from previous studies [20]; [17]. Kajiado County is subdivided into 5 subcounties, namely, Kajiado North, Kajiado South, Kajiado East, Kajiado West, and Kajiado Central. Kajiado County experiences both long and short rains. The pattern of the rainfall is not uniform across the county. Rainfall ranges between 300 mm and 1250 mm.

### 2.2. Determining the Seroprevalence of *Taenia saginata* Cysts (*Cysticercus bovis*) in Cattle Presented for Slaughter in Selected Slaughterhouses in Kajiado County, Kenya

#### 2.2.1. Study Design

A cross-sectional study design was used to collect data from selected slaughterhouses in Kajiado County.

#### 2.2.2. Study Sites

Kitengela, Kiserian, and Keekonyokie slaughterhouses were selected because they receive animals from the entire county and are category B slaughterhouses (have a high daily throughput). They are also located in different subcounties; the Kitengela slaughterhouse is in Kajiado East Subcounty and Kiserian and Keekonyokie slaughterhouses are in Kajiado West Subcounties.

#### 2.2.3. Sample Size Determination

Sample size was determined according to the method described in [21]. The total sample size of cattle is 38. The sample size was adjusted to 150 using design effect to cater for possible clustering (aspect of different subcounties) effects.

#### 2.2.4. Blood Collection and Serum Preparation

Each selected slaughterhouse was visited daily for twenty days. Only animals that originated from within the county were sampled. During the visits, sampling was done after the jugular was slit, whereby approximately 10 ml of blood was collected from each animal. The sample collection bottles were labelled and placed in a cool box that contained icepacks. The blood samples were transported to the Department of Public Health Pharmacology and Toxicology Laboratory of the University of Nairobi in a cool box within two hours of blood collection. The blood samples were kept overnight at room temperature and centrifuged at 3000 g for 30 minutes. Serum was then extracted into cryovials, labelled, and stored at −20°C until used for serology.

#### 2.2.5. Serology

Serum samples from cattle were screened for circulating *Taenia* antibodies using LSY-30021 cysticercus (Cyt) antibody ELISA test kit for livestock. The procedure was adopted from the manufacturer, Shenzhen Lvshiyuan Biotechnology Co., Ltd., China. The antigens used in seroprevalence were cysticercus antigen from cattle serum precoated on the ELISA plates.

### 2.3. Determining the Annual Hospital Reported Cases of *Taenia saginata* in Humans Attending Level 4 Hospitals in Kajiado County, Kenya

The study utilized retrospective data of patients who visited all level 4 hospitals between 2015 and 2021. Those that were diagnosed with *Taenia saginata* were noted and recorded. The data were obtained from all level 4 hospitals in Kajiado County. In Loitokitok Sub-County Hospital and Kajiado County Referral Hospital, the data were obtained by perusing available records. In Kitengela Sub-County Hospital, Ongata Rongai Sub- County Hospital, and Ngong Sub-County Hospital, the data were obtained from the District Health Information Software (DHIS-2) system.

### 2.4. Assessing the Risk Factors for *Taenia saginata* in Humans in Kajiado County, Kenya

#### 2.4.1. Study Population

Livestock keeping households in Kajiado County consist of both male and female livestock keepers. The households to be interviewed were randomly selected based on the following inclusion criteria: (i) permanent resident of Kajiado County, (ii) a pastoralist household, and (iii) a household that keeps cattle.

#### 2.4.2. Sampling

Multistage sampling was used to sample respondents; whereby from Kajiado County, all subcounties were selected, and from each subcounty, one ward was selected using simple random sampling and random number generator. The selected ward served as the site for conducting interviews. A sample size of 140 was used based on the method mentioned in [22]. The study involved conducting interviews in at least 25 households from each ward, until sample saturation was obtained.

#### 2.4.3. Data Collection

Interviews were used to collect quantitative and qualitative data using structured questionnaires. Two sets of interviews were conducted with the livestock keepers and with the meat inspectors. Digitized questionnaires using the Open Data Kit (ODK) application were administered to livestock keepers and used to collect data on meat inspection and preparation, animal husbandry practices, availability, and access to anthelmintic and management practices for taeniasis. Informed consents were sought before conducting the interviews amongst livestock keepers. The local leaders were informed of the purpose and scope of the study for community support. Meat inspectors were interviewed using questionnaires to collect data on the number of positive cases of *Taenia saginata* cysts that were observed between June and July 2021.

#### 2.4.4. Pretesting the Questionnaire

This was conducted in Keekonyokie ward of Kajiado West Subcounty. 10% of the sample size was used for the pretest, which is equivalent to 14 respondents.

#### 2.4.5. Conducting the Interviews

The interviews were conducted in selected wards in each subcounty as follows: IIdamat in Kajiado Central, Kitengela in Kajiado East, Magadi in Kajiado West, Ngong in Kajiado North, and Kimana in Kajiado South.

### 2.5. Data Management and Analysis

Data on the number of serum samples that tested positive and negative for circulating antibodies were recorded in Microsoft Excel, and prevalence was calculated as the number positive for circulating antibodies divided by the total number of serum samples that were analyzed and presented as a percentage.

Test for proportions was used to assess for differences between/among the stratified prevalence (by slaughterhouse).

Data from hospital records on the number of patients diagnosed with taeniasis between 2015 and 2021 and the total number of patients that visited the facility between 2015 and 2021 were recorded in Microsoft Excel, and yearly proportion of hospital records of taeniasis was calculated as the number of patients that were diagnosed with taeniasis divided by the total number of patients that visited the facility.

Test for proportions was used to assess for the differences between/among the stratified proportions (by subcounty and by year).

Further comparison of the proportions was conducted using the pairwise test for proportions to detect if the differences were statistically significant.

Responses from the questionnaires on risk factors for human taeniasis were exported to Excel for data validation and cleaning. Data analysis was done using STATA version 17 to determine the frequency distribution for the different risk factors. The results are presented in tables.

Data analysis was performed using R software version 4.2.1 to assess the difference between/among the stratified proportions of reported hospital taeniasis cases (by year and by subcounty).

### 2.6. Ethical Consideration

The study was reviewed, and ethical approval was granted by the University of Nairobi, Faculty Biosafety Animal Use and Ethics Committee, and a research permit from National Commission of Science, Technology, and Innovation was granted. Authorizations to conduct the study in Kajiado County were obtained from the County Director of Veterinary Services and County Director of Health. All interview respondents were provided with written consents, and all the respondents that consented to be part of the study filled in the questionnaire for livestock keepers and questionnaire for meat inspectors.

## 3. Results

### 3.1. Seroprevalence of *Taenia saginata* Cysts (*Cysticercus bovis*) in Cattle Presented for Slaughter in Selected Slaughterhouses in Kajiado County, Kenya

From 150 serum samples that were analyzed, 4 tested positive for *Taenia saginata* cysts (*C. bovis*). This translates to an overall prevalence of 2.67% at 95% confidence interval (0.89%, 7.1%; *p* value <0.0001) in the selected slaughterhouses, as presented in Table 1. The highest prevalence at 4% and 95% confidence interval (0.69%, 14.86%) was encountered at the Kitengela slaughterhouse, followed by the Keekonyokie slaughterhouse, 3.33%, at 95% confidence interval (0.58%, 12.55%). No positive sample was recorded from the Kiserian slaughterhouse. The difference in seroprevalence between Keekonyokie (3.33%) and Kitengela (4%) slaughterhouses was not statistically significant at 95% confidence interval (−0.07, 0.08; *p* value −1).

### 3.2. Annual Hospital Reported Cases of *Taenia saginata* in Humans Attending Level 4 Hospitals in Kajiado County, Kenya

#### 3.2.1. Proportion of Human Taeniasis Recorded in Level 4 Hospitals in Kajiado County between 2015 and 2021

Data from hospital records showed that out of a total of 1,487,687 patients that visited different facilities between 2015 and 2021, 29 were diagnosed with human taeniasis, as indicated in Table 2. The highest proportion (0.006%) was encountered in 2015 and the least proportion (0.004%) in 2020.

There was an overall statistically significant difference in the proportion of recorded cases of *T. saginata* in level 4 hospitals (*p* value 0.004) across the different years. Pairwise comparison further revealed a significantly higher proportion in 2015 compared to 2016 (*p* value 0.025), 2015 compared to 2019 (*p* value 0.009), and 2015 compared to 2020 (*p* value 0.007).

### 3.3. Risk Factors for *Taenia saginata* in Kajiado County, Kenya

#### 3.3.1. Demographic Characteristics of the Respondents

140 respondents participated in the questionnaire survey. Majority of the respondents about 94 (67%) were males and 46 (33%) were females. On the age group segregation, the majority, 67 (48%) were between 31 and 60 years, followed by the youth, 18−30 years, 65 (46%), and the elderly (˃60 years) about 8 (6%) were the minority.

#### 3.3.2. Risk Factors for Human Taeniasis

Risk factors considered for this study included home slaughter and meat inspection, preparation and consumption of beef, and presence, and use of latrines reported presence of tapeworm infection and treatment. These are presented in Table 3.

Home slaughter, consumption of parts of beef raw, presence and use of latrines, and treatment were statistically significant (*p* value <0.5) variables for taeniasis, as indicated in Table 3.

Home slaughter was significantly high in all subcounties except in Kajiado North Subcounty where only a minority 9 (35%) did slaughter at home. In all the subcounties, all respondents indicated that they still consume some parts of beef raw. In terms of meat preparation, more than half of the respondents indicated they consume improperly cooked beef in Kajiado West and Kajiado South. In the other subcounties, a majority indicated that they consume beef that has been properly cooked/roasted. Presence and use of latrines were significantly high across all the subcounties. All subcounties except Kajiado South indicated low presence of taeniasis in the past two years. All subcounties reported that when people get infected with taeniasis, they seek treatment.  Table 4 shows the risk factors by subcounties.

#### 3.3.3. Presence of *Cysticercus bovis* in Cattle

The meat inspectors did not record any cases of *C. bovis* between June and July 2021 in the selected slaughterhouses.

## 4. Discussion

*Taenia saginata* presents both public health and economic importance in developed and developing countries. The occurrence of the larval stage of this parasite, *Cysticercus bovis*, may vary from place to place depending on the standards of meat inspection, cattle husbandry practices, level of hygiene, and methods of sewage management. From the slaughterhouse survey in this study, the seroprevalence of *Taenia saginata* cysts (*C. bovis*) in cattle in Kajiado County between June and July 2021 was 2.67% among 150 cattle that were sampled. The ELISA method adopted for this study was testing for the presence of circulating *Taenia* antibodies. It, therefore, can be rightly inferred that all the cattle that had positive ELISA test results had previous exposure to eggs of *Taenia saginata.*

The seroprevalence results observed in this study seem to agree with the questionnaire survey results that indicated that 21% of Kajiado locals reported to have seen people releasing proglottids. In addition, a run through the hospital records also showed that cases of human taeniasis are being reported in level 4 hospitals in Kajiado County. It is, therefore, quite evident that active transmission of *Taenia saginata* could be occurring in Kajiado County.

However, during the study period, the meat inspectors in all the sampling abattoirs did not find any case of *C. bovis*. This apparent contradictory results, as compared to the ELISA test and the outcome of the community administered questionnaire, may be due to the fact that the sensitivity of visual meat inspection, however well it may be undertaken, could be as low as 40% [18], while the sensitivity of the ELISA test is as high as 80% [18]. Although a positive antibody ELISA test is not a confirmation of a *C. bovis* infection in the tested animals, the results are still epidemiologically significant as they indicate exposure of the cattle to *T. saginata* eggs, and by extension, the existence of transmission risk factors of the food-borne parasite infection.

The findings of meat inspection results as reported by meat inspectors confirm that the sensitivity of meat inspection in detecting *Taenia saginata* cysts (*Cysticercus bovis*) is much lower than that of ELISA [11]; [19]; [12]; [8]; [23]; [18]; [24]. The findings of this study show that the antibody ELISA test for *C. bovis* inspection was three times more sensitive than visual meat inspection. As pointed out by [18], the high sensitivity of the ELISA test for *C. bovis* compared to visual meat inspection makes it a tool of choice for *T. saginata* epidemiological studies [18].

Prevalence of *C. bovis* using meat inspection has previously been reported in Kajiado County. In 1981, a retrospective study utilizing national database on abattoir records noted a prevalence of 10.3% in Ngong District of Rift Valley Province (currently part of Kajiado County). The records were obtained from the Director of Veterinary Services Office in Kabete [20]. In 2016, a study conducted in slaughterhouses in Kajiado County noted a prevalence of 2.56% for bovine cysticercosis, using meat inspection [17]. In the current study, the meat inspectors from all the study slaughterhouses reported no case of *C. bovis* during the study period, translating to a prevalence of 0%. These three studies looked at together seem to indicate a progressive reduction in the prevalence of *C. bovis* over a forty-year period. This is also in agreement with anecdotal reports from meat inspectors and the University of Nairobi lecturers who have been taking students to slaughterhouses for meat inspection practicals and have indicated that there has been a drastic reduction in *C. bovis* between 1980(s) and the current year.

In addition, reported cases of human taeniasis from all the level 4 hospitals in Kajiado County revealed that the proportion of cases of *Taenia saginata* also seemed to have declined between 2015 and 2021. A total of 29 patients were diagnosed with human taeniasis between 2015 and 2021 out of 1,487,687 patients who visited different facilities. The highest annual average proportion (0.006%) was encountered in 2015 and the least proportion (0.004%) in 2020. The highest proportions were recorded from the only two hospitals located in the more urbanized areas of Rongai and Ngong. This may be explained by the fact that urban hospitals have better diagnosis and improved facilities, and people in the urban areas consume more beef than the rural people who depend on milk and meat from sheep and goats. In addition, the two subcounties are residence to people from diverse regional backgrounds that will tend to seek treatment in the health facilities whenever they fall ill.

The likely factors that may have led to the apparent reduction in cases of *Cysticercus bovis* and *Taenia saginata* over the years are varied, and it may be difficult to identify which of them has had the greatest impact in reducing the risk of infection. These factors could include the increasing general level of education, increased use of anthelmintics, and increased availability and use of latrines.

Increased general level of education can be alluded for construction of more schools and introduction of adult education over time. The current literacy levels in Kajiado County stands at 70% [25], from very low levels amongst eligible children as shown by different studies [26–28]. The introduction of a hygiene programme (WASH) in 2013 also ensured that women and school going children do not walk long distances in search of water and in turn led to an increase in enrollment of children in schools. With enhanced general education comes enhanced hygiene awareness that would include knowledge of taeniasis and cysticercosis, and the affected humans would easily take medication whenever they release proglottids.

It is also noted that over time anthelmintics have become available and easily accessible even at the local shops in the study area. This was confirmed by the current study that noted that 89% of respondents take drugs (anthelmintics) to get rid of *Taenia saginata* eggs and proglottids. The anthelmintics are sourced from hospitals, chemists, local shops, and even from traditional medicine men. In some instances, the dewormers are distributed to school going children by the Ministry of Health to help in prevention and control of the tapeworms. Control measures have also been affected, which include construction and use of latrines. The current study noted that most households had and used latrines. The WASH program that was introduced in 2013 also promoted reduction in open defecation through advocating for construction of latrines [29].

However, despite the noted reduction in the prevalence of *T. saginata* in Kajiado County, it has also been shown that transmission of this disease continues howbeit at a reduced level. This situation is not surprising as this study has shown that at least three risk factors persist at some significant level within the community. The questionnaire respondents indicated that uninspected home slaughter, eating of raw/improperly cooked meat, and open defecation by the herders still pose a risk to humans getting taeniasis and subsequently cattle getting cysticercosis.

Home slaughter is still being practiced in Kajiado County. 90% of those who slaughter at home indicated that the meat is not inspected by a veterinary meat inspector. This is in line with the findings of [17, 30, 31] that established that *Taenia saginata* has been reported where the community practice uninspected home slaughter. 100% of the respondents indicated that they consume some parts of beef raw, while 47% indicated that they consume improperly cooked beef. This is significant in the spread of taeniasis to humans, since the cysts can lodge anywhere and not necessarily in the predilection sites, due to variation in distribution of cysticerci to preferred sites [19]. Most homes would slaughter sheep and goat regularly and occasionally slaughter cattle during major functions. This, therefore, means that most of the beef/parts of beef consumed raw or improperly cooked are sourced from slaughterhouses and butcheries which are mainly concentrated in the peri-urban areas. This would explain why more cases of human taeniasis were reported in Kajiado North Subcounty which has the level 4 facilities based in periurban areas.

Use of latrines aids in proper disposal of fecal matter, thus significantly reducing contamination of the farms, grasses, and pastures with taenia eggs. This breaks the cycle of transmission of taenia eggs and proglottids from humans to the cattle. This study indicates that education and the WASH project have helped in creating knowledge, therefore increasing construction and use of latrines. A study that was conducted in 2017 in Kajiado County noted a significant lack of latrines that played a role in spread of parasite egg [17]. A study by [32] indicated that 59.2% of households in Kajiado East, West, South, and Central Subcounties do not have access to toilets and 98.4% of that population practice open defecation. In as much as a higher percentage that have and use latrines, there are herders who move with livestock from place to place and do not use latrines. They practice open defecation and therefore distribute the taenia eggs and proglottids, posing a challenge in the control and prevention of human taeniasis.

This study also reported that 21% of residents have seen people infected with *Taenia saginata* in the past two years. This is an indication that there exists an active presence of infection in the community even though at low levels as indicated by records from level 4 hospitals. The meat inspectors did not record any positive cases of *Taenia saginata* cysts (*C. bovis*) during the study period, as per their responses from the questionnaires. The absence of *Cysticercus bovis* at meat inspection could indicate that the risk of infection in humans is low. The respondents indicated that taenicides are available and easily accessible, and whenever a person is infected, they can easily obtain the drugs. The drugs are majorly sourced from chemists. They are also available in hospitals and in local shops. Most respondents self-medicate and, therefore, do not visit the hospitals unless they have a serious disease. This could explain why the prevalence of taeniasis from hospital records was low.

From the seroprevalence of *C. bovis* levels reported, proportion of humans that were diagnosed with taeniasis in level 4 hospitals and risk factors assessed, it is apparent that *Taenia saginata* is still present in Kajiado County, though at low levels and uninspected home slaughter, consumption of raw/improperly cooked beef and open defecation by herders still pose a risk to humans getting infected by *Taenia saginata.* However, the presence and use of latrines and availability, accessibility, and use of taenicides have significantly contributed to reduction of *T. saginata*. Considering the cultural practices of the local community (carrying out home slaughter and consumption of raw meat and transhumant nature of moving with livestock in search of water and pasture by herders) and since humans are the only definitive hosts of *Taenia saginata*, control of the infection should be centered towards continuous focused education coupled with regular deworming of human carriers and especially the herders and school going children. This will gradually result in behavior and culture change that will ultimately reduce the prevalence and lead to elimination of the disease.

## 5. Conclusions and Recommendations

In conclusion, *Taenia saginata* and *Cysticercus bovis* are still present in Kajiado County but at comparatively much lower prevalence than previously reported. The study confirms that serology (ELISA) is three times as sensitive as meat inspection, and the risk of humans getting infected with *Taenia saginata* is significantly reduced by decreased bovine cysticercosis, increased use of latrines, and availability of drugs for treating taeniasis and that uninspected home slaughter and consumption of raw/improperly cooked beef still pose a risk of humans getting infected with taeniasis in Kajiado County.

Based on the above conclusions, it was recommended that there is a need for more sustained community health education on upscaled one health approach in managing cysticercosis and taeniasis to reduce impact in both cattle and humans. This can be done through improved and focused awareness creation on the zoonotic importance of *Taenia saginata* and the need to break the transmission cycle in cattle and humans.

## Figures and Tables

**Table 1:** Seroprevalence of *Taenia saginata* cysts (*Cysticercus bovis*) in selected slaughterhouses.

Subcounty	Slaughterhouse	Total sampled	Total positive	Prevalence in %	95% confidence interval	*p* value
Kajiado West	Kiserian	40	0	0	(0, 0)	—
Kajiado West	Keekonyokie	60	2	3.33%	(0.58%, 12.55%)	<0.0001
Kajiado East	Kitengela	50	2	4%	(0.69%, 14.86%)	<0.0001
	OVERALL	150	4	2.67%	(0.89%, 7.1%)	<0.0001

**Table 2:** Proportion of human taeniasis recorded in level 4 hospitals in Kajiado County between 2015 and 2021.

Year	Total number that attended all the hospitals	Number positive	Proportion (%)	95% confidence interval
2015	178,839	10	0.006	(0.003%, 0.04%)
2016	202,086	2	0.0009	(0.0002%, 0.004%)
2017	137,890	4	0.003	(0.0009%, 0.008%)
2018	240,844	5	0.002	(0.0008%, 0.005%)
2019	292,810	3	0.001	(0.0003%, 0.003%)
2020	211,211	1	0.0004	(0.00002%, 0.003%)
2021	224,007	4	0.0018	(0.00057%, 0.0049%)

**Table 3:** Risk factors for human taeniasis.

Variable	Yes, *n* (%)	No, *n* (%)	*p* value
Home slaughter	105 (75%)	35 (25%)	<0.0001
Consumption of beef raw (some parts)	140 (100%)	0 (0%)	<0.0001
Preparation (properly cooked)	74 (53%)	66 (47%)	0.5329
Presence and use of a latrine	124 (89%)	16 (11%)	<0.0001
Reported presence of *T. saginata* infection	29 (21%)	111 (79%)	<0.0001
Treatment	124 (89%)	16 (11%)	<0.0001

**Table 4:** Risk factors for human taeniasis by subcounties.

Subcounty variable	East, *N* = 22	West, *N* = 39	North, *N* = 26	South, *N* = 26	Central, *N* = 27	Total = 140	*p* value
Home slaughter	17 (77%)	32 (82%)	9 (35%)	25 (96%)	22 (81%)	105	<0.0001
Consumption of raw beef (some parts)	22 (100%)	39 (100%)	26 (100%)	26 (100%)	27 (100%)	140	—
Preparation (improperly cooked)	8 (36%)	24 (62%)	1 (4%)	24 (92%)	9 (33%)	66	<0.0001
Presence of latrine	22 (100%)	35 (90%)	25 (96%)	16 (62%)	26 (96%)	124	<0.0001
Presence of *T. saginata*	5 (23%)	4 (10%)	1 (4%)	16 (62%)	3 (11%)	29	<0.0001
Treatment	21 (95%)	34 (87%)	26 (100%)	21 (81%)	22 (81%)	124	0.1179

## Data Availability

The data used to support the findings of this study are available from the corresponding author upon request.
